# Immediate loading of short implants: A systematic review

**DOI:** 10.34172/japid.2021.002

**Published:** 2021-03-06

**Authors:** Mahdi Hadilou, Pooya Ebrahimi, Behnaz Karimzadeh, Ashkan Ghaffary, Leila Gholami, Zahra Fathifar

**Affiliations:** ^1^Student Research Committee, Faculty of Dentistry, Tabriz University of Medical Sciences, Tabriz, Iran; ^2^Dental Implants Research Center, Department of Periodontology, School of Dentistry, Hamadan University of Medical Sciences, Hamadan, Iran; ^3^School of Health, Tabriz University of Medical Sciences, Tabriz, Iran

**Keywords:** Immediate loading, Short implants, Single implants

## Abstract

**Background:**

This systematic review aimed to determine the effectiveness and outcomes of immediate loading methods for short dental implants.

**Methods:**

The authors independently conducted an electronic search in the PubMed, Embase, EBSCO, ProQuest, and Cochrane databases for relevant articles published until November 15, 2020. The references of the included studies were assessed, and a manual search was conducted in Google Scholar and PubMed to find additional relevant studies.

**Results:**

Finally, three studies were selected and included in this systematic review. Significant heterogeneity existed in the design of the included studies, and due to the low number of the included studies, the authors could not perform a meta-analysis. The studies showed that the survival rate of immediate-loaded short implants is comparable to conventional loading Methods. However, more marginal bone loss is expected. Overall, the immediate loading of short dental implants might be clinically successful.

**Conclusion:**

Based on the results, immediate loading protocols might be safely used for short implants. However, caution should be exercised in interpreting these results.Future welldesigned randomized clinical trials with more participants and study power are necessary to support the findings of this systematic review.

## Introduction

Tooth loss has been associated with physiological and psychological problems for patients, such as alterations in diet,^1,2^ tooth drifting and tipping,^3^ emotional effects,^4,5^ and opposite tooth overeruptions.^6^ Lost teeth can be replaced with various methods, including bridges, partial dentures,^7^ and dental implants.^8^ Dental implants are excellent replacements for lost teeth, with studies suggesting an approximate 95% success rate in 15 years.^9^ However, since their introduction into dentistry as a viable option for tooth replacement, they have undergone changes to provide patients with more comfort.^10^ Implants with standard sizes have been considered more successful in previous studies than implants with shorter lengths, generally due to their stability.^11^ However, normal-sized implants cannot be placed in any region as they require a specific amount of bone and might interfere with vital organs, such as maxillary sinuses or the inferior alveolar nerve. Therefore, other procedures might be necessary, like bone grafting or sinus lifting, resulting in an increased chance of surgical complications, including infections, inferior alveolar nerve damage, and sinus perforations.^12^

The application of shorter implants in areas with special conditions, such as regions with markedly low bone levels, can help reduce the number of interventions needed to place an implant in the resorbed alveolar bone.^12^ Like the implant size, their loading schedule also has to be altered to shorten patients’ treatment period to increase patient satisfaction.^13^ As expected, these changes to conventional procedures of implant placements entail more caution to achieve satisfactory outcomes.^10^

In the case of markedly resorbed residual ridges and elderly patients, immediate loading methods are demanded at a higher rate nowadays.^14,15^ Immediate loading is the placement of restorative material within two days of implant surgery.^16^ It can be undertaken if adequate primary stability is gained for an implant fixture. The minimum primary stability or torque needed to follow immediate loading protocols is 35 Ncm.^17^ Immediate loading protocols are best in situations where the patient cannot tolerate two-stage surgeries or multiple visits; also, improved esthetics, enhanced function, and comfort are expected.^18,19^ Short implants are beneficial in areas with a low residual bone height, especially in cases that we cannot use bone augmentation protocols due to financial, age-related, or anatomic issues.^19-21^ In the anterior maxillary segment, conventional implant loading increases patient worries about aesthetic results in this region. Improvements in implant materials, designs, and surface textures have allowed clinicians to proceed with immediate loading and function in special cases.^22^

This study examined the prognosis of the immediate loading of short implants by conducting a systematic review of previous studies on the immediate loading of short implants simultaneously to help clinicians offer the best options to their patients by assessing the durability, survival rate, and patient satisfaction.

## Methods

### Focused question

The Preferred Reporting Items for Systematic Review and Meta-Analyses (PRISMA) guidelines were followed.^23^

The addressed PICO was: “Can we efficiently use immediate loading protocols for short implants to achieve satisfactory osseointegration and functional results for patients with limited residual bone?”

### Selection criteria

The eligibility criteria for this systematic review followed the PICOS question:

- Population: The participants’ ages >18 years, including males and females who were candidates for single tooth replacement with short implants (<8 mm in length)^21,24^ with immediate loading protocols.
- Interventions: The intervention group in the studies should have undergone a short implant placement surgery with immediate loading protocols.
- Outcomes: The current review aimed to cover as many outcomes related to implant success as possible. These outcomes consisted of implant survival rate, marginal bone loss, and implant mobility. Mentioned outcomes were the main concerns of the review; however, other outcomes studied in the articles were also included as the secondary outcomes.

### Study design

Only randomized controlled trials (RCTs) were included as eligible studies. Studies containing animal studies, in vitro studies, retrospective and cohort studies, review articles, unpublished studies, and articles in languages other than English were excluded. Articles in which the patients had a systemic disease and studies assessing overdentures, splinted implants, bridges, ridge augmentation, sinus lifting, and application of normal-sized implants were also excluded.

### Search strategy

The authors (ZF and MH) independently conducted an electronic search in the databases of PubMed, Embase, EBSCO, ProQuest, and Cochrane for related articles published until November 15, 2020. References of the included studies were assessed, and a manual search was conducted in Google Scholar and PubMed to find additional relevant studies. The search strategy was as follows:

((((“Dental Implants”[Mesh]) OR (“Dental Implantation, Endosseous”[Mesh]))) AND (short implant)) AND ((((“Immediate Dental Implant Loading”[Mesh]) OR (Early Dental Implant Load*)) OR (conventional load*)) OR (delayed load*))

### Screening methods and data extraction

Two reviewers (BK and AG) independently screened the studies in three stages. First, duplicate articles were found and removed. Then, titles and abstracts were examined according to the eligibility criteria. After that, full texts of articles that met the eligibility criteria were selected. If there were any disagreements between the two reviewers, a third reviewer (LG) decided whether to include the study or not.

Data were extracted from the included studies in two separate tables containing the following parameters: author/year, study type,country, the number of subjects and their mean age, use of antibiotic prophylaxis, the system, number, diameter and height of the implants, type and site of the procedure, type of occlusal contact, time of provisional crown, time of the final crown,whether flap reflection or bone grafting was carried out or not, evaluated criteria and study outcomes, insertion torque, and follow-ups.

### Risk of bias in individual studies

To determine the validity of the included RCTs, the authors assessed the risk of bias associated with random sequence generation, allocation concealment, blinding of participants and personnel, blinding of outcome assessment, incomplete outcome data, selective reporting, and other biases.

The risk of bias of studies was assessed according to Cochrane Handbook for Systematic Reviews of Interventions^25^ as follows: “high risk of bias” (-) in red, “low risk of bias” (+) in green, or “unclear risk of bias” (U) in yellow for each of these sections.

## Results

### Study selection

**Figure 1:**
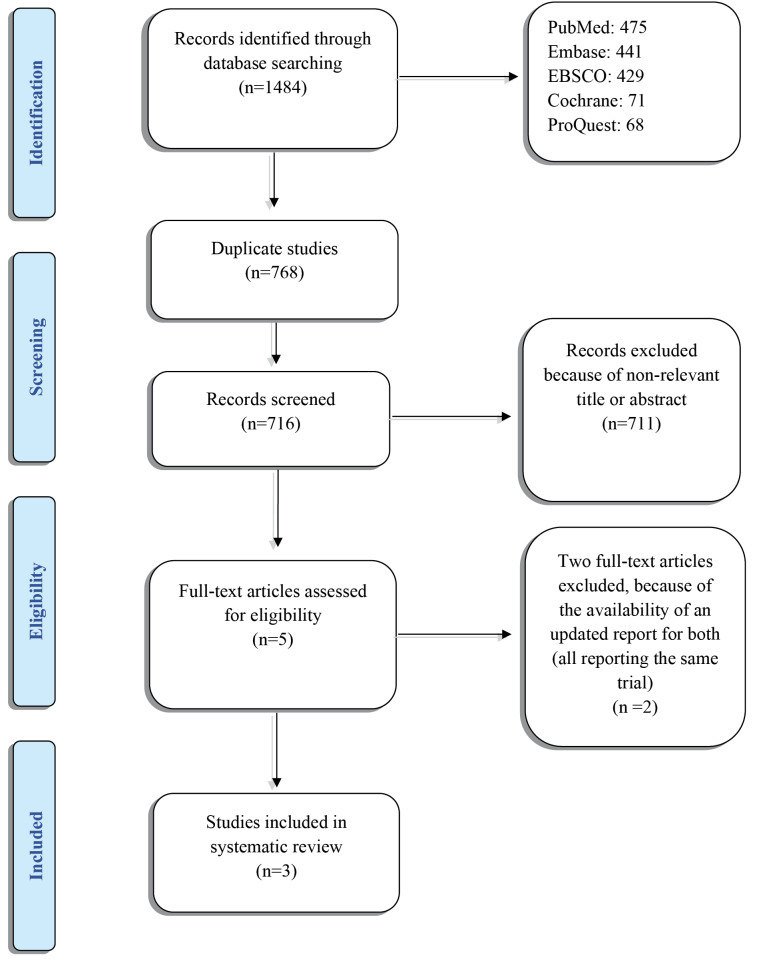
Flow diagram according to preferred reporting items for systematic reviews and meta-analysis (PRISMA).

Initially, 1484 studies were found in the search. After the duplicate articles were removed (n=768) and titles and abstracts were rescreened, 711 articles did not meet the eligibility criteria of the review and were excluded. Five full-text papers were selected for the screening, of which two papers were excluded^26,27^ because of the availability of an updated report for both (all reporting the same trial).^28^ The final selection resulted in the inclusion of three studies (Tables 1 and 2).^28-30^ Figure 1 shows the flow diagram of the study selection process and results of the literature search according to PRISMA guidelines.^23^

**Table 1:** General characteristics of studies

**Study**	**Year**	**Country**	**Study type**	**No. of participants**	**Mean** **age**	**Antibiotic prophylaxis**	**Implant characteristics**			
							**System**	**Number (excluded)**	**Diameter**	**Height**
Weerapong et al^30^	2018	Thailand	RCT	46	50.50 (20-61)	NM	PW+ Dental Implant System, Thailand	23 (4)	NM	6 mm
Ayna et al^29^	2018	Germany	RCT	63	54.68 ± 8.63	Amoxicillin 875 mg + clavulanic acid 125 mg were given 1 h before surgery, and two times a day for six days thereafter	Internal-hexed self-tapping titanium implants with large grit, Sand-blasted and acid-etched surfaces (LGI plus, Hi-Tec Implant Ltd. Herzliya Israel)	48	5, 6 mm	6 mm
Cannizzaro et al^28^	2018	Italy	RCT	60	35 (18-57)	Amoxicillin 2 g 1 hour before the intervention. Patients allergic to penicillin were given clarithromycin 500 mg 1 hour before the intervention.	NanoTite parallel-walled dental implants (Biomet 3i, Palm Beach, FL, USA)	29(1)	4, 5, 6 mm	6.6 mm

Abbreviations: RCT, randomized controlled trial, NM, not mentioned.

**Table 2:** Details of intervention in each study

**Study**	**Implant Site/No.**	**Follow-up time**	**Occlusal contact**	**Time of provisional crown (after implant placement)**	**Time of definitive crown**	**Healed or fresh socket**	**Flap reflection**	**Insertion Torque (Newton-Centimeter)**	**Bone grafting**
Weerapong et al^30^	Mandibular molar teeth	1, 2, 3, 4 weeks postsurgery 2, 4 months and 1 year	Centric occlusion/ eccentric occlusion avoided	Immediately after surgery	NM	Healed	No	>35 Ncm (42.61 ±7.52)	No
Ayna et al^29^	Maxillary molar teeth	Up to 5 years after implant placement	Slight centric occlusion/ eccentric occlusion avoided	NM	3 months after implant placement	NM	Yes	≥35 Ncm	No
Cannizzaro et al^28^	Both mandibular and maxillary sites were included 17 maxilla 12 mandible	Up to 9 months after implant placement	Slight occlusal contact with the opposing dentition	Immediately after surgery/ within a few hours	3 months after implant placement	9 fresh sockets	8 elevated flaps	>40 Ncm	In the presence of a residual gap between the implant surface and the bone wall ≤1.5 mm, the gap was filled with Bio-Oss.

### Characteristics of the included studies

**Figure 2:**
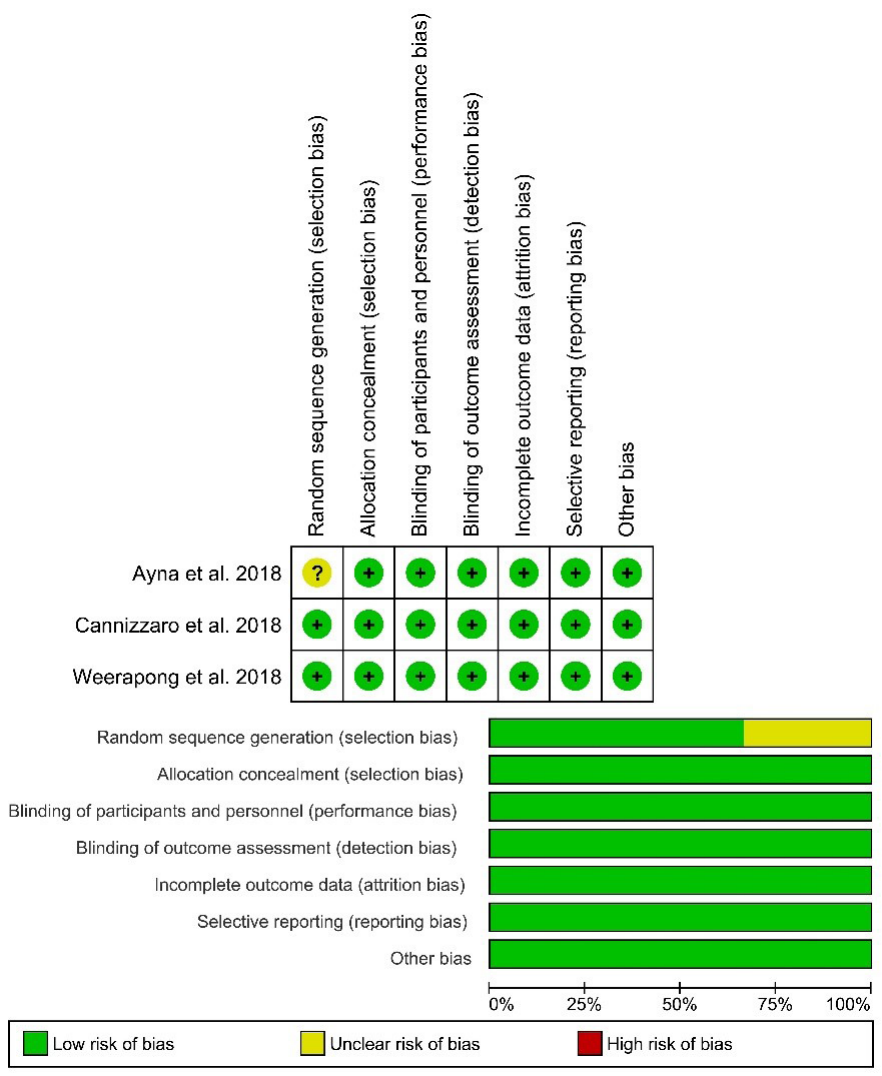
Risk of bias analysis.

Among the three selected studies,all were categorized as RCTs. These trials were conducted in Italy,^28^ Germany,^29^ and Thailand.^30^ The age range of participants in the included studies was 46‒63. Two studies^28,29^ used antibiotic prophylaxis before the procedures. The lengths of the placed implants were 6 mm in two studies^29,30^ and 6.6 mm in the other one^28^. The number of implants ranged from 23 to 48. The site of placement was also different among studies. Implants were placed in the maxilla by Ayna et al^29^ and in the mandible by Weerapong et al.^30^ Cannizzaro et al placed implants in both the mandible and maxilla.^28^ The follow-up periods varied from nine months to five years. All the study designs avoided occlusal contacts on eccentric movementsand used provisional crowns immediately or shortly after implant placement.The surgery method in one study was flapless,^30^ and in the other two studies, flap surgery was performed.^28,29^ In all of the studies, the insertion torque was not less than 35 Ncm,and one study used bone grafting techniques in the presence of a gap between the surface of the implant and the bone wall.^28^ Figure 2 presents the risk of bias summary. Overall, there was a low risk of bias in all the included studies; except for one issue about randomization of patients in the study by Ayna et al.^29^

### Main outcomes of the studies

All the included studies suggested that the immediate-loaded short implants can be clinically successful in the short and long term, and it is possible to achieve clinically successful outcomes in immediate loading protocols. Ayna et al^29^ pointed out that the immediate loading of implants increased bone loss and bleeding on probing, with a statistically significant difference.

The marginal bone loss and the survival rates in the study by Weerapong et al^30^ were not significantly different between conventional and short implants. However, Ayna et al^29^ showed that bone loss was significantly lower in delayed loading than in the immediately loaded implants. However, the survival rates were not significantly different. Cannizzaro et al^28^ reported that bone loss was significantly lower in the delayed loading than the immediate loading. In this study, the survival rates were not significantly different.

## Discussion

In the present report, we exclusively analyzed clinical trials regarding the immediate loading of short dental implants regardless of the follow-up time.

Weerapong et al^30^ studied the immediate loading of mandibular molar implants and found that the survival rate, stability, and marginal bone loss in the immediate-loaded implants were not statistically different from what it is in conventional methods.

Ayna et al^29^ studied the immediate loading protocols in the maxillary molar area and found that the clinical results were satisfactory. However, increased bone loss and bleeding on probing levels were observed in the immediate loading group compared to the delayed methods. This might be due to the low quality of bone in the posterior maxilla or other less likely factors, like the torque of insertion or the operator’s skill.

Cannizzaro et al^28^ placed dental implants in both maxillary and mandibular areas by a flapless method, immediately loaded them, followed the patients for nine years, and concluded that immediate placement of short implants could be clinically successful in the long term.

Regarding the advantages of the immediate loading of short implants, the time of treatment is significantly shortened, and the prosthetic treatment can be as good as the conventional methods. However, single-tooth dental implants have been reported to have a higher chance of clinical failure.^31^ Immediate-loaded dental implants preserve the structural integrity of peri-implant soft tissues with the provisional restoration during the healing period.^32^ Concerning marginal bone loss and implant survival rate, the loading protocol is thought to be not relevant in the clinical success.^33^

A review suggested no significant difference between conventional and immediate loading protocols for conventional (normal-sized) implants,^34^ and this study also confirmed it about short implants. However, another review suggested that clinicians should be cautious about using immediate loading protocols for dental implants in the single-tooth restorations in the anterior maxilla because of probable marginal bone remodeling and gingival changes.^35^

In the included studies, the survival rate of the immediate-loaded short implants was not statistically different from the control groups. Also, other studies support this finding.^36,37^ Overall, the survival rate of immediate-loaded short implants in the mandible is higher than the maxilla.^36^

It is believed that the high stability of implants immediately after the placement has a significant role in implant success rate, allowing immediate loading protocols.^38^ Conventionally and immediately loaded implants had the same success rate and marginal bone loss when they were inserted with adequate torque (>20-45 Ncm).^39^ The studies included in this study met this requirement.^28-30^

Conventional implant insertion techniques require elevation of full-thickness soft tissue flaps. However, the flapless technique is considered better because it does not compromise the vascular supply of peri-implant tissues, resulting in less marginal bone loss.^40^ In this review, one study^30^ used a flapless protocol, and the other two studies^28,29^ used the traditional approach.

There is still controversy about whether the immediate loading of dental implants should be non-occlusal or occlusal. A meta-analysis demonstrated no association between this and bone loss or implant success rates.^41^ In our review, all the studiesused immediate provisional restorations with occlusal centric contacts, with no occlusal contacts in eccentric movements to establish undisturbed healing.

### Limitations of the study

Several outcome measures have been used in dental implant research. However, there are no standard criteria for the assessment of outcomes.^42,43^ The number of studies on replacing teeth using short dental implants with immediate functional loading protocols is limited.^37,44,45^ The main limitation of the present study was that we did not include studies in which the patients had certain risk factors, such as smoking and diabetes. Patients who needed alveolar ridge augmentation before implant placement were excluded since it can act as a confounding factor when assessing only the effects of length of short implants. Also, due to incomplete information about long-term follow-ups and methods in the included studies and the limited number of included studies, the authors could not perform a meta-analysis. Because of the limited number of RCTs, this review’s results should be interpreted with caution. This study aimed at precisely predefined aspects of implants that play a crucial role in the therapy’s success; however, some aspects of implants were not included in this review because they were not mentioned in the included studies and should be examined in future studies, such as different implant placement protocols, and the condition of the bone and the soft tissue during the implant placement period.

Furthermore, well-designed studies with similar methodological design and loading criteria, with larger sample sizes and long-term follow-ups are necessary to draw evidence-based conclusions for clinical decision making.

## Conclusions

Within the limitations of this review, the authors concluded that immediate loading protocols for placing short implants might be safe, with no significant difference between conventional and immediate loading protocols regarding implant success rates. However, more RCTs with larger sample sizes and long-term follow-ups are necessary for better decision-making. Therefore, clinicians should be very cautious.

## Authors’ Contributions

Design of the study protocol: MH; searching in the databases: ZF, and MH; screening of the studies: BK, AG, and LG; risk of bias assessment: BK, and MH; interpretation of data: LG, and PE; drafting the work: PE, and MH; revision: LG. All authors read and approved the final manuscript.

## Availability of data

The data from the reported study are available upon request from the corresponding author.

## Ethics approval

Not applicable.

## Competing interests

The authors declare no conflicts of interest related to the publication of this study.
